# Associations between the Planetary Health Diet in Late Pregnancy and Pregnancy Outcomes in Women with a Raised BMI: Insights from the PEARS Study

**DOI:** 10.1016/j.cdnut.2026.109540

**Published:** 2026-09-19

**Authors:** Aoife Davis, Sophie Callanan, Gillian A Corbett, Eileen C O’Brien, Alexander P Douglass, Fionnuala M McAuliffe

**Affiliations:** 1 UCD Perinatal Research Centre, UCD School of Medicine, University College Dublin, National Maternity Hospital, Dublin, Ireland; 2 National Maternity Hospital, Dublin, Ireland; 3 School of Biological, Health and Sports Sciences, Technological University Dublin, Dublin, Ireland; 4 College of Health and Agricultural Sciences, University College Dublin, Dublin, Ireland

**Keywords:** pregnancy, planetary health, maternal pregnancy outcomes, neonatal outcomes, sustainable diets

## Abstract

**Background:**

Diet quality in pregnancy is a modifiable factor with important implications for maternal and neonatal outcomes. The Planetary Health Diet (PHD), designed to promote both human health and environmental sustainability, has not been comprehensively evaluated in high-risk obstetric populations.

**Objectives:**

This study aimed to examine the PHD in a pregnant cohort with a raised body mass index (BMI) and its associations with pregnancy outcomes, hypothesizing that greater adherence is associated with more favorable pregnancy outcomes.

**Methods:**

This was a secondary analysis of data from the pregnancy exercise and nutrition research study with smartphone application support study. Pregnant women (*n* = 319) with a BMI ≥25 kg/m^2^ an 11 d ≤39.9 kg/m^2^ in early pregnancy were included in the analysis, and the cohort was dichotomized into “Low” (≤92.9) and “High” (>92.9) PHD Index (PHDI) groups based on the median score in late pregnancy (∼28 wk gestation). Differences in pregnancy outcomes between the “High” and “Low” groups were explored, with results adjusted for multiple testing.

**Results:**

In adjusted models, a “High” PHDI score was associated with a lower odds of preeclampsia and/or pregnancy-induced hypertension [adjusted odds ratio (OR): 0.21; 95% confidence interval (CI): 0.06, 0.68; *P* = 0.01), and a higher likelihood to initiate breastfeeding postpartum (adjusted OR: 1.84; 95% CI: 1.04, 3.24; *P* = 0.04). In the adjusted model, those in the “High” PHDI group had a lower risk of delivering an infant born small for gestational age (SGA) (birthweight <10th centile) (OR: 0.31; 95% CI: 0.10, 1.93, *P* = 0.04).

**Conclusions:**

: This diet is associated with lower odds of hypertensive outcomes among pregnant women with a raised BMI, while promoting more sustainable food choices. Greater adherence to the PHD is also associated with a lower risk of delivering an infant born SGA and higher breastfeeding initiation rates, highlighting its potential benefits beyond pregnancy.

## Introduction

Pregnancy outcomes for both mother and child are influenced by various environmental factors, with maternal nutrition playing a key role in creating a favorable environment for fetal development [1]. Maternal overweight and obesity (OVOB) during pregnancy are recognized risk factors for adverse outcomes during pregnancy, including gestational diabetes mellitus (GDM), hypertensive conditions, preeclampsia, labor complications, and pregnancy loss or spontaneous miscarriage [[2], [3], [4], [5]]. These conditions also negatively impact neonatal outcomes, contributing to increased rates of preterm delivery, neonatal morbidity and mortality, as well as a higher likelihood of delivering large-for-gestational-age infants [[5], [6], [7]].

Pregnancy is a period of heightened motivation for positive lifestyle changes, making it an ideal window for promoting healthy dietary habits. Different maternal dietary patterns, such as the Mediterranean diet, Alternate Healthy Eating Index for Pregnancy, and Dietary Approaches to Stop Hypertension, have been associated with favorable outcomes for both maternal and neonatal health [1,[8], [9], [10], [11]]. However, evidence of dietary interventions resulting in improved pregnancy outcomes is conflicting.

When curating dietary guidelines aimed at protecting maternal health, it is also essential to consider the environmental impact of different food choices [12]. Given the potential of the food system to enhance public health and foster environmental sustainability, a diet that benefits both planetary health and pregnancy outcomes is extremely important [13]. The Planetary Health Diet (PHD) is a dietary model designed to promote global health and reduce the environmental impact of food systems [14]. It prioritizes the consumption of plant-based foods, with limited intake of animal products.

To assess adherence to this diet, the PHD Index (PHDI) was developed as a novel tool to quantify alignment with the PHD’s guidelines [15]. Although the PHD has been recognized for its potential role in preventing chronic diseases such as cardiovascular disease, diabetes, and cancer, its specific impact on pregnancy outcomes remains largely unexplored [16,17]. Emerging evidence has linked higher PHD adherence to reduced risk of low birthweight and preterm birth, along with higher intakes of key pregnancy nutrients [12,18,19].

Most existing research in pregnant populations with OVOB has focused narrowly on outcomes such as gestational weight gain (GWG), GDM, or birthweight, without evaluating a broader range of maternal and neonatal health outcomes [16,17]. This exploratory analysis addresses the critical gap by investigating the relationship between adherence to the PHD in late pregnancy and a comprehensive set of maternal and neonatal outcomes in a high-risk cohort of women with OVOB. We hypothesize that high adherence to the PHD in late pregnancy may reduce risk of adverse pregnancy and neonatal outcomes in a population of women with OVOB.

## Methods

### Study design

This is a secondary analysis of data obtained from the pregnancy exercise and nutrition research study with smartphone application support (PEARS) randomized controlled trial (RCT). The trial was designed to reduce risk of GDM among women with OVOB (ISRCTN29316280). The PEARS trial was conducted in the National Maternity Hospital, Dublin, Ireland between 2013 and 2016. Full details of the study design and protocol have been published previously [20]. Details on eligibility criteria and the intervention for the PEARS study are provided in Supplemental Methods. Briefly, women were enrolled if they were between 18 and 45 y of age, at 10–18 wk gestation with a singleton pregnancy, had a BMI between 25 and 39.9 at recruitment, and possessed a smartphone [20]. The exclusion criteria for the PEARS trial included women with a history of GDM, any pre-existing medical conditions, multiple pregnancy, women <18 or >45 y of age, fetal anomaly, previous stillbirth/perinatal death, and/or insufficient English. The multicomponent behavioral intervention was a low glycemic index diet, exercise prescription, supported with a specifically designed smart phone application aiming to reduce GDM. No differences in GDM rates were noted, and the intervention group had less GWG and improved nutritional intakes.

### Ethical approval

The PEARS trial received ethical approval from The National Maternity Hospital Ethics Committee in October 2012 and was carried out in accordance with the Declaration of Helsinki of 1975 as revised in 1983. Informed written consent was obtained from all participants.

### Maternal characteristics

Maternal weight was recorded at baseline (14–16 wk), and 28- and 34-wk study visits. Height was recorded at baseline and BMI (kg/m^2^) was calculated. Maternal age, gestation at baseline, parity, ethnicity, education level, smoking status, and supplement use were collected from medical records or self-reports in the lifestyle questionnaire. Participant addresses were obtained from medical records and used to calculate the Pobal Haase Pratschke (HP) deprivation index [21].

### Dietary assessment in late pregnancy

Dietary data in late pregnancy were obtained using 3-d food diaries completed at ∼28 wk gestation. Participants from both trial arms were asked to record their intake as accurately as possible including 2 weekdays and 1 weekend day to assess normal eating habits. Details on dietary assessment are provided in Supplemental Methods. Dietary underreporting was evaluated using the Goldberg method, which involves analyzing the ratio of reported energy intake to estimated basal metabolic rate (BMR) [22,23]. An energy intake to BMR ratio ≤0.9 was classified as underreporting, whereas ratios above 0.9 were considered indicative of plausible reporting [24].

### Assignment of PHDI scores

Individual PHDI scores were calculated for each participant based on dietary data obtained at 28 wk gestation. The specific scoring methodology used was chosen because of its robust methodology. Detailed methodology about the scoring methods has been described previously [12,15]. In brief, PHDI scores were derived from self-reported intake of 14 food groups, with scoring thresholds based on the midpoint of the recommended ranges from the EAT-Lancet Commission scientific report and validated by Bui et al. [25]. The 14 components were classified as either adequacy components, which are encouraged for consumption or moderation components, which are discouraged or subject to recommended limits. Higher scores indicate greater compliance with dietary recommendations. For the present analysis, participants were dichotomized based on the median PHDI score in early pregnancy into a “Low” (≤92.9) and “High” (>92.9) PHDI group [15]. For details on the calculation of the PHDI scores, see Supplemental Methods.

### Pregnancy outcome data

Data on maternal and neonatal pregnancy outcomes were obtained from hospital records postdelivery. For details on measurements and cutoff points, see Supplemental Table 1. Because of the small sample size and low frequency of hypertensive disorders, the occurrence of preeclampsia and/or pregnancy-induced hypertension (PIH) was combined into a single binary variable (“Preeclampsia and/or PIH”) for regression analyses.

Maternal outcomes include preeclampsia (yes/no), PIH (yes/no), maternal weight before delivery (kg), GWG (kg), GWG criteria (adequate, inadequate, and excessive), anemia in late pregnancy (hemoglobin levels <11 g/dL), and placental weight (g). Neonatal outcomes include preterm delivery of <37 wk or ≥37 wk, head circumference (cm), birthweight (g), low birthweight (≤2.5 kg), macrosomia (cutoffs; >4 kg, ≥4.5 kg), birthweight centile, small for gestational age (SGA) (birthweight <10th centile), large for gestational age (birthweight >90th centile), and the Gestation Network’s Bulk Calculator 6.2.3 United Kingdom was used to calculate birth weight centiles [20]. Breastfeeding initiation after delivery was grouped as any exposure to breast milk or formula only. More details on pregnancy outcome data are provided in Supplemental Table 1.

### Statistical analysis and justification of sample size

Participants were eligible for inclusion in this analysis if they had a PHDI score in late pregnancy and ≥1 nonmissing pregnancy outcome. This led to a total sample size of 319 (Figure 1). As this was a secondary analysis requiring complete dietary data in late pregnancy, there is potential for selection bias. Statistical analyses were performed using SPSS version 29.0 for Mac (Macintosh). Normality of all continuous variables was evaluated through conceptual understanding of the data, visual inspection of histograms, and analysis using Kolmogorov–Smirnov tests. Data were presented as mean and SD for normally distributed variables, whereas non-normally distributed variables were reported using the median and IQR (25th, 75th percentiles). Categorical variables were presented as number and frequency [*n* (%)]. When comparing maternal baseline characteristics, nutritional status, maternal pregnancy outcomes, and neonatal outcomes between participants in the “High PHDI” and “Low PHDI” groups, independent *t*-tests were used for normally distributed variables, Mann–Whitney U tests for skewed data, and chi-square tests for categorical variables.

**FIGURE 1:**
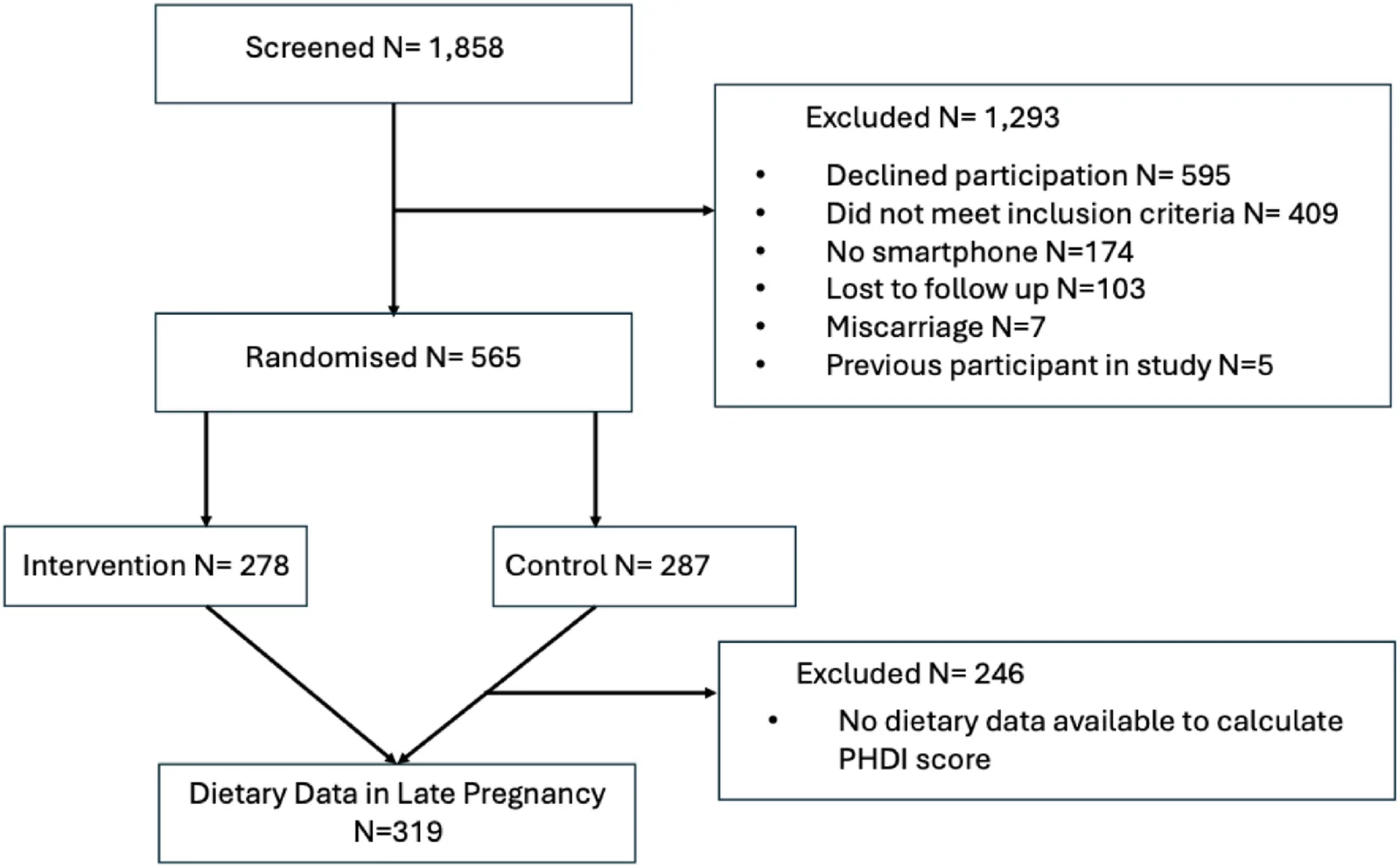
Flow diagram of the PEARS study. PEARS, pregnancy exercise and nutrition research study with smartphone application support; PHDI, Planetary Health Diet Index.

Linear regression analyses were conducted to explore the relationship between PHDI scores and late pregnancy nutrient intake. Both linear and logistic regression models examined the relationship between PHDI scores and pregnancy outcome data. Both unadjusted (crude) and adjusted regression models were created using a forced entry approach method, comparing the “High PHDI” and “Low PHDI” groups, and analyzing PHDI as a continuous variable. The PHDI was analyzed as a continuous variable to preserve information and improve precision displayed as “Per 1-point increment in PHDI score,” representing the change in risk associated with each 1-point increase in an individual’s PHDI score.

Because this was a secondary analysis of the PEARS trial, no primary outcome was prespecified. The pregnancy outcomes examined are listed in Table 1. Of the 319 women with a late-pregnancy PHDI score, sample sizes for all maternal and fetal outcomes were lower and varied between outcomes (*n* ranging from 107 to 317), according to data collection and availability at the time, with all efforts made to include as much information as possible from existing records. The number of missing pregnancy outcomes is outlined in Table 1.

**TABLE 1:** Maternal and neonatal outcomes for total cohort

	*n*	*n* with missing data	*n* (%)/mean (SD)/median (IQR)
**Maternal outcomes**			
**Preeclampsia (yes)**^1^	282	37	8 (2.5%)
**PIH**^1^	283	36	10 (3.1%)
**Preeclampsia and/or PIH**^1^	282	37	18 (6.4%)
**Final maternal weight before delivery**^2^**(kg)**	207	112	89 (82.6, 99.3)
**Gestational weight gain**^3^**(kg)**	204	115	11.86 (5.60)
**GWG criteria**^1^	204	115	
**Adequate**			33 (16.2%)
**Inadequate**			54 (26.5%)
**Excessive**			117 (57.4%)
**Anemia in late pregnancy (yes)**^1^	107	212	24 (22.4%)
**Placental weight**^3^**(g)**	271	48	658.85 (146.55)
**Neonatal outcomes**			
1**Gestation at delivery**^1^	314	5	
**<37 wk**			11 (3.5%)
**≥37 wk**			303 (96.5%)
**Head circumference**^2^**(cm)**	286	33	35 (34, 36)
**Birthweight**^3^**(g)**	317	2	3629.64 (517.17)
**Low birthweight (≤2.5 kg)**^1^	317	2	6 (1.9%)
**Macrosomic (>4 kg)**^1^	317	2	76 (24%)
**Macrosomic (≥4.5 kg)**^1^	317	2	12 (3.8%)
**Birthweight centile**^2^	302	17	51.4 (27.83, 74.98)
**Birthweight <10th centile (SGA)**^1^	302	17	18 (6.0%)
**Birthweight >90th centile (LGA)**^1^	302	17	32 (10.6%)
**Breastfeeding initiation after delivery**^1^	315	4	
**Any breast milk exposure**			222 (70.5%)
**Formula**			93 (29.5%)

The combined preeclampsia and/or PIH variable reflects participants with complete data for both outcomes.

Abbreviations: GWG, gestational weight gain; LGA, large for gestational age; PIH, pregnancy-induced hypertension; SGA, small for gestational age.

1 Categorical variables, expressed as *n* (% denominator).

2 Non-normal distribution, expressed as median [interquartile range (IQR)].

3 Normal distribution, expressed as mean (SD).

Adjusted regression analysis was also performed to examine the association between each individual PHDI component and any significant pregnancy outcomes per 1-point increment in each component score. Two models were analyzed for each outcome: Model 1 adjusted for study allocation group (intervention, yes/no); Model 2 adjusted for study allocation group (intervention, yes/no), maternal BMI (continuous kg/m^2^) at baseline (14–16 wk gestation), physical activity [continuous metabolic equivalents of task (METs)/d], and HP index (continuous score) for preeclampsia/PIH and SGA outcomes, and additionally included education status for breastfeeding initiation.

To assess potential selection bias, pregnancy outcomes were compared between participants from the original PEARS study with complete dietary data included in the analysis and those with missing dietary data who were excluded. Continuous variables were compared using Mann–Whitney U or independent samples *t*-tests, and categorical variables using chi-square tests.

Confounding variables were selected before the analysis based on existing literature and consensus [3,26,27]. When investigating maternal and neonatal outcomes, Model 1 was adjusted for the study allocation group (intervention, yes/no). Model 2 accounts for potential confounders including study allocation group (intervention, yes/no), maternal BMI (continuous kg/m^2^) at baseline (14–16 wk gestation), physical activity (continuous METs/d), and HP index (continuous score). MET activity was recorded as minutes for week, and details on the specific calculation have been described previously [28]. For analyses related to breastfeeding initiation postpartum, maternal education status (third-level education yes/no) was also included as a covariate because of its potential influence on this outcome [29]. Dietary reporting status (plausible reporter yes/no) was additionally adjusted for in nutrient intake analyses only and not included in models assessing pregnancy outcomes. Full data were available for all confounders apart from late METs, where complete data were available for 314 of the 319 participants. Pairwise deletion was applied to missing data, as most variables had complete observations. All statistical tests were 2-tailed, and significance was determined at *P* < 0.05.

Because of the large number of statistical tests performed (*n* = 340), the Benjamini–Hochberg correction was applied to manage the false discovery rate (FDR) at 20%. Raw *P* values were ranked in ascending order and converted to FDR-adjusted *q* values, with *q* values ≤0.20 considered statistically significant. Initially, results in a *P* value of <0.05 were considered significant, after which *P* values were converted to *q* values to identify truly significant results. As a sensitivity analysis, associations were also examined against a more conservative 10% FDR threshold. To formally test for nonlinear associations between the PHDI score and significant maternal and neonatal outcomes, curve estimation analyses were performed comparing linear, logarithmic, quadratic, and cubic functional forms.

## Results

### Sample size

Figure 1 describes the population selection for this analysis. A total of 565 women were randomly assigned in the PEARS study, with 319 providing dietary data in late pregnancy from which PHDI scores could be calculated. Of the 319 women with PHDI scores, all had ≥1 pregnancy outcome recorded.

### Maternal characteristics

Table 2 presents the maternal characteristics of the total cohort as well as the differences in maternal characteristics between the “High PHDI” and “Low PHDI” groups. Those in the “High PHDI” group had a significantly lower BMI. A significantly higher proportion of the “High PHDI” group received the intervention compared with the “Low PHDI” group. Comparable trends were observed after Benjamini–Hochberg adjustment for multiple testing. Maternal and neonatal outcomes for the total cohort are described in Table 1, including the number of participants with complete data and the extent of missing data for each outcome. The frequencies of adverse outcomes and continuous measures are summarized.

**TABLE 2:** Maternal characteristics of the total cohort at baseline (12–16 wk gestation)

	Total cohort		*n* with missing data	Low PHDI (≤92.9)		High PHDI (>92.9)		*P* value
	*n*	Median (IQR)/*n* (%)		*n*	Median (IQR)/*n* (%)	*n*	Median (IQR)/*n* (%)	
**PHDI score**^1^	319	92.90 (85.72, 100.50)	0	160	85.77 (80.84, 89.62)	159	100.50 (96.13, 105.70)	<0.001
**Age (y) at recruitment**^2^	319	32.55 (4.33)	0	160	32.13 (4.72)	159	32.98 (3.87)	0.08
**Weight (kg) at recruitment**^1^	319	76.70 (71.00, 85.60)	0	160	78.55 (72, 88.78)	159	76.20 (70.20, 82.30)	0.02
**BMI (kg/m^2^) at recruitment**^1^	319	28.24 (26.7, 31.02)	0	160	28.88 (27.01, 31.80)	159	27.59 (26.33, 30.19)	0.003
**BMI category**								
**BMI**^3^**<30 kg/m^2^**	319	220 (69.0%)		160	102 (63.7%)	159	118 (74.2%)	0.04
**BMI**^3^**≥30 kg/m^2^**	319	99 (31.0%)		160	58 (36.3%)	159	41 (25.8%)	
**HP Pobal Index**^1^	319	7.1 (-2.1, 14.2)	0	160	5.2 (-2.9, 13.48)	159	8.2 (-1.0, 14.5)	0.22
**Parity (multiparous)**^3^	317	48 (15.1%)	2	159	27 (17%)	158	21 (13.3%)	0.36
**Ethnicity (White-Irish)**^3^	305	240 (78.7%)	14	154	126 (81.8%)	151	114 (75.5%)	0.70
**Tertiary-level education**^3^	319	212 (66.5%)	0	160	101 (63.1%)	159	111 (69.8%)	0.21
**Group**			0					0.009
**Control**^3^	319	160 (50.2%)		160	92 (57.5%)	159	68 (42.8%)	
**Intervention**^3^	319	159 (49.8%)		160	68 (42.5%)	159	91 (57.2%)	
**Supplements (yes)**								
**Folic acid pre-pregnancy**^3^	183	95 (51.9%)	136	95	45 (47.4%)	88	50 (56.8%)	0.20
**Folic acid since conception**^3^	183	178 (97.3%)	136	95	93 (97.9%)	88	85 (96.6%)	0.59
**Other supplements prior**^3^	183	35 (19.1%)	136	95	16 (16.8%)	88	19 (21.6%)	0.41

Maternal BMI categories as per the WHO BMI classification.

Abbreviations: HP, Haase and Pratschke Social Deprivation Index; PHDI, Planetary Health Diet Index.

1 Non-normal distribution, compared using Mann–Whitney U test. Expressed as median [interquartile range (IQR)].

2 Normal distribution, compared using independent samples *t*-tests. Expressed as mean (SD).

3 Categorical variables, compared with chi-square test. Expressed as *n* (% denominator). *P* value <0.05 is statistically significant.

### Associations between the PHDI score and maternal outcomes in pregnancy

Table 3 presents the associations between the PHDI score in late pregnancy and maternal outcomes, comparing the “High PHDI” and “Low PHDI” groups. For each outcome, it reports the raw frequencies in both groups alongside crude {Model 1, adjusted for study allocation group (intervention, yes/no) and fully adjusted [Model 2, adjusted for study allocation group (intervention, yes/no)], maternal BMI (continuous kg/m^2^) at baseline (14–16 wk gestation), physical activity (continuous METs/d), and HP index (continuous score)} odds ratios (ORs) or beta coefficients with 95% confidence intervals (CIs) and *P* values. Results are presented both for the binary PHDI grouping and for PHDI as a continuous variable (per 1-point increment). A total of 18 cases of preeclampsia and/or PIH were recorded in this population. The only statistically significant finding was for the combined preeclampsia and/or PIH outcome, where the “High PHDI” group had substantially lower odds in both the crude and adjusted models (adjusted OR: 0.21; 95% CI: 0.06, 0.68; *P* = 0.01; *q* = 0.06), with a consistent effect observed per 1-point PHDI increment (adjusted OR: 0.94; 95% CI: 0.89, 0.99; *P* = 0.01; *q* = 0.06). Results remained significant after adjusting for multiple testing; however, given the small number of events, effect estimates are imprecise, and these findings should be regarded as exploratory. Additional analyses to test for nonlinear associations for each significant outcome yielded similar results (Supplemental Table 2).

**TABLE 3:** Associations between the Planetary Health Diet Index in late pregnancy and maternal pregnancy outcomes

Maternal outcomes										
	Low PHDI (≤92.9)		High PHDI (>92.9)		*P* value	Model	High PHDI		Per 1-point increment in PHDI score	
	n	n (%)/median (IQR)	n	n (%)/median (IQR)			Odds ratio/ß (95% CI)^1^	*P* value	Odds ratio/ß (95% CI)^1^	*P* value
**Preeclampsia (yes)**^2^	138	8 (5.0%)	144	0 (0%)	0.01	—	—	—	—	—
**PIH (yes)**^2^	140	6 (3.8%)	143	4 (2.5%)	0.54	—	—	—	—	—
**Preeclampsia and/or PIH (yes)**	139	14 (10.1%)	143	4 (2.8%)	0.01	Model 1^3^	0.24 (0.08, 0.76)	0.02	0.94 (0.90, 0.99)	0.02
						Model 2^4^	0.21 (0.06, 0.68)	0.01	0.94 (0.89, 0.99)	0.01
**Final maternal weight before delivery (kg)**	100	90.65 (82.85, 102.15)	107	88.3 (82.4, 96.3)	0.13	Model 1^3^	−2.76 (−6.15, 0.64)^1^	0.11	−0.09 (−0.25, 0.07)^1^	0.28
						Model 2^4^	−0.87 (−3.53, 1.78)^1^	0.52	−0.01 (−0.13, 0.12)^1^	0.90
**GWG (kg)**	99	12.09 (5.37)	105	11.65 (5.83)	0.58	Model 1^3^	−0.31 (−1.89, 1.27)^1^	0.70	−0.04 (−0.11, 0.04)^1^	0.35
						Model 2^4^	−0.60 (−2.20, 1.01)^1^	0.46	−0.05 (−0.12, 0.03)^1^	0.22
**GWG criteria**^2^										
**Adequate**	99	13 (13.1%)	105	20 (19%)	0.50	—	—	—	—	—
**Inadequate**		28 (28.3%)		26 (24.8%)		---	---	---	---	---
**Excessive**		58 (58.6%)		59 (56.2%)		---	---	---	---	---
**Anemia in late pregnancy (yes)**	56	10 (17.9%)	51	14 (27.5%)	0.24	Model 1^3^	0.61 (0.24, 1.55)	0.30	0.98 (0.94, 1.03)	0.49
						Model 2^4^	0.58 (0.23, 1.51)	0.27	0.98 (0.94, 1.03)	0.43
**Placental weight (g)**	137	650 (560, 737.50)	134	650 (568.75, 756)	0.87	Model 1^3^	−1.75 (−37.59, 34.09)^1^	0.92	−0.25 (−1.94, 1.45)^1^	0.78
						Model 2^4^	−0.72(-37.31, 35.87)^1^	0.97	−0.22 (−1.95, 1.52)^1^	0.81

The combined preeclampsia and/or PIH variable reflects participants with complete data for both outcomes.

Abbreviations: CI, confidence interval; GWG, gestational weight gain; PIH, pregnancy-induced hypertension.

1 Odds ratio/ß (95%) represents “High PHDI” group compared with “Low PHDI” group. *n* for each outcome reflects participants with complete data for that specific measure. Sample sizes vary for each outcome, and outcomes were collected from different sources, including hospital records, self-report questionnaires, postnatal visits, and availability differed by outcome.

2 Regression analysis could not be completed because of low numbers.

3 Model 1 is adjusted for the allocation group (intervention, yes/no).

4 Model 2 is adjusted for study allocation group (intervention, yes/no), maternal BMI (continuous kg/m^2^) at baseline (14–16 wk gestation), physical activity [continuous metabolic equivalents of task (METs)/d], and HP index (continuous score).

### Associations between the PHDI score and neonatal outcomes in pregnancy

After controlling for confounders and multiple testing, infants born to mothers in the “High PHDI” group were at lower risk of being born SGA [13 (8.6%) compared with 5 (3.3%); *P* = 0.04; *q* = 0.17] (Table 4). This association also remained significant when PHDI score was treated as a continuous variable. Those in the “High PHDI” group were also significantly more likely to initiate breastfeeding. After controlling for study allocation group (intervention, yes/no), maternal BMI (continuous kg/m^2^) at baseline (14–16 w gestation), physical activity (continuous METs/d), HP index (continuous score), tertiary-level education status (yes/no), and multiple testing, the crude and adjusted models suggested that a “High PHDI” was associated with a higher likelihood to initiate breastfeeding (adjusted OR: 1.84; 95% CI: 1.04, 3.24; *P* = 0.04; *q* = 0.17). Similar results were found per 1-point increase in PHDI score (adjusted OR: 1.03; 95% CI: 1.0, 1.06; *P* = 0.04; *q* = 0.17) (Table 4). Additional analyses to test for nonlinear associations for each significant outcome yielded similar results (Supplemental Table 2).

**TABLE 4:** Associations between the Planetary Health Diet Index in late pregnancy and neonatal outcomes

Neonatal outcomes										
	Low PHDI (≤92.9)		High PHDI (>92.9)		*P* value	Model	High PHDI		Per 1-point increment in PHDI score	
	*n*	*n* (%)/median (IQR)/mean (SD)	*n*	*n* (%)/median (IQR)/mean (SD)			Odds ratio/ß (95% CI)^1^	*P* value	Odds ratio/ß (95% CI)^1^	*P* value
**Gestation at delivery**	158		156		0.78					
**<37 wk**		6 (3.8%)		5 (3.2%)		Model 1^2^	1.10 (0.32, 3.75)^1^	0.88	1.01 (0.95, 1.07)^1^	0.81
**≥ 37 wk**		152 (96.2%)		151 (96.8%)		Model 2^3^	1.12 (0.32,3.92)^1^	0.87	1.01 (0.95,1.07)^1^	0.87
**Head circumference (cm)**	144	35 (34.33, 36.4)	142	35 (34, 36)	0.13	Model 1^2^	−0.32 (−0.69, 0.05)^1^	0.10	−0.01 (−0.02, 0.01)^1^	0.49
						Model 2^3^	−0.28 (−0.65, 0.10)^1^	0.15	−0.004 (−0.02, 0.01)^1^	0.69
**Birthweight (g)**	159	3638.30 (536.67)	158	3620.93 (498.33)	0.77	Model 1^2^	−13.54 (−130.36, 103.29)^1^	0.82	3.52 (−2.0, 9.03)^1^	0.21
						Model 2^3^	0.54 (−117.77, 118.85)^1^	0.99	4.21 (−1.38, 9.80)^1^	0.14
	159		158							
**Low birthweight ≤2.5 kg**		3 (1.90%)		3 (1.90%)	0.99	Model 1^2^	1.00 (0.2, 5.13)	1.0	1.02 (0.94, 1.10)	0.71
						Model 2^3^	1.09 (0.2, 5.86)	0.92	1.02 (0.94, 1.11)	0.63
**Macrosomia birthweight >4 kg**		37 (23.3%)		34 (21.5%)	0.71	Model 1^2^	0.95 (0.55, 1.63)	0.85	1.01 (0.98, 1.04)	0.53
						Model 2^3^	0.96 (0.55, 1.68)	0.89	1.01 (0.98, 1.04)	0.50
**Macrosomia birthweight ≥4.5 kg**		6 (3.80%)		6 (3.80%)	0.99	Model 1^2^	1.12 (0.35, 3.59)	0.85	1.02 (0.97, 1.08)	0.44
						Model 2^3^	1.18 (0.36, 3.86)	0.79	1.02 (0.97, 1.08)	0.45
**Birthweight centile**	151	54.40 (27.20, 76.30)	151	50.10 (27.90, 74.40)	0.83	Model 1^2^	0.11 (−6.34, 6.56)^1^	0.97	0.21 (−0.09, 0.52)^1^	0.17
						Model 2^3^	0.36 (−6.21, 6.93)^1^	0.92	0.22 (−0.09, 0.54)^1^	0.16
**Birthweight <10th centile (SGA)**	151	13 (8.6%)	151	5 (3.3%)	0.05	Model 1^2^	0.35 (0.12, 1.03)	0.06	0.94 (0.90, 0.99)	0.01
						Model 2^3^	0.31 (0.10, 0.93)	0.04	0.93 (0.89, 0.98)	0.01
**Birthweight >90th centile (LGA)**		18 (11.9%)		14 (9.3%)	0.46	Model 1^2^	1.07 (0.48, 2.36)	0.87	1.0 (0.97, 1.04)	0.90
						Model 2^3^	1.05 (0.47, 2.34)	0.91	1.0 (0.97, 1.04)	0.89
**Breastfeeding initiation after delivery**	159		156		0.003					
**Any BM exposure**		100 (62.9%)		122 (78.2%)		Model 1^2^	2.21 (1.32, 3.67)	0.002	1.04 (1.02, 1.07)	0.001
**Formula**		59 (37.1%)		34 (21.8%)		Model 2^4^	1.84 (1.04, 3.24)	0.04	1.03 (1.0, 1.06)	0.04

Abbreviations: CI, confidence interval; LGA, large for gestational age; PHDI, Planetary Health Diet Index; SGA, small for gestational age.

1 Odds ratio/ß (95% CI) represents “High PHDI” group compared with “Low PHDI” group. *n* for each outcome reflects participants with complete data for that specific measure. Sample sizes vary for each outcome, and outcomes were collected from different sources, including hospital records, self-report questionnaires, postnatal visits, and availability differed by outcome.

2 Model 1 is adjusted for the study allocation group (intervention, yes/no).

3 Model 2 is adjusted for study allocation group (intervention, yes/no), maternal BMI (continuous kg/m^2^) at baseline (14–16 wk of gestation), physical activity [continuous metabolic equivalents of task (METs)/d], and HP index (continuous score).

4 Model 2 is additionally adjusted for education status (third-level education yes/no).

Both the binary group comparison and the continuous per-point associations with preeclampsia/PIH remained significant at both 10% and 20% FDR thresholds. The adjusted associations with SGA and breastfeeding initiation in the “High PHDI” group met a 20% but not a 10% FDR threshold. The continuous, per 1-point increments association between PHDI score and SGA was robust to either threshold. The equivalent continuous association for breastfeeding initiation only met the 20% FDR threshold. Given the secondary, exploratory nature of this analysis, all findings should be interpreted with caution.

### Nutrient intake

Descriptive overview of nutrient intake in late pregnancy is provided in Supplemental Tables 3–5 with detailed overview of the differences in nutrient intake between the “High PHDI” and “Low PHDI” groups. Several differences were observed between the “High PHDI” and “Low PHDI” groups, notably compared with those in the “Low PHDI” group, those in the “High PHDI” group had higher intakes of ω-6, fiber, vitamin C, magnesium, iron, zinc, and lower intakes of vitamin D, vitamin B3, vitamin B12, and selenium. Comparable trends were observed after Benjamini–Hochberg adjustment for multiple testing.

### PHDI score component analysis

No individual PHDI component was independently associated with preeclampsia and/or PIH after adjustment (Supplemental Table 6). Higher wholegrain scores were associated with lower odds of delivering an infant SGA (OR: 0.88; 95% CI: 0.77, 1.0; *P* = 0.04). For breastfeeding initiation, higher scores for several PHDI components were associated with a greater likelihood of initiating breastfeeding. Specifically, in adjusted models, higher whole fruit scores (OR: 1.10; 95% CI: 1.01, 1.19; *P* = 0.02), higher nonstarchy vegetable score (OR: 1.25; 95% CI: 1.10, 1.42; *P* < 0.001), higher starchy vegetable score (OR: 1.13; 95% CI: 1.03, 1.24; *P* = 0.01), and higher fish score (OR: 0.93; 95% CI: 0.86, 1.0; *P* = 0.04).

### Missing data analysis

There were no statistically significant differences in any pregnancy outcome between those with and without dietary data, including rates of preeclampsia and/or PIH (6.7% compared with 6.4%; *P* = 1.0), SGA (8.1% compared with 6.0%; *P* = 0.38), or breastfeeding initiation (67.3% compared with 70.5%; *P* = 0.45) (Supplementary Table 7).

## Discussion

Greater adherence to the PHD in late pregnancy was associated with lower odds of a combined hypertensive outcome (preeclampsia and/or PIH) in a cohort of women living with OVOB, although this association was based on a small number of cases and should be interpreted cautiously. Women with a higher PHDI score had a lower risk of delivering an infant born SGA and were more likely to initiate breastfeeding. These findings contribute to the growing discussion on the benefits of sustainable diets and their potential role in supporting maternal and neonatal health. However, as this was an exploratory analysis, these findings should be interpreted cautiously and considered hypothesis-generating rather than confirmatory.

Our findings indicate distinct differences in maternal pregnancy outcomes between those with a “Low PHDI” score compared with those with a “High PHDI” score. Few studies have investigated the relationship between adherence to specific dietary patterns and pregnancy outcomes. Most existing studies focused on the Mediterranean diet, which has been inversely linked to various adverse pregnancy outcomes, including PIH, preeclampsia, preterm delivery, and intrauterine growth restriction or the dietary inflammatory index, which has been similarly associated with such outcomes [[30], [31], [32]].

Previous research has linked lower intake of plant-derived foods with a higher risk of hypertensive disorders in pregnancy, including preeclampsia [33]. This aligns with our findings, where women in the “Low PHDI” group, who consumed fewer plant-based foods, accounted for most hypertensive cases in our sample, although the small number of events limits the strength of this conclusion. The exact mechanism of the beneficial effects of a plant-based diet on blood pressure is unknown; however, the relationship between higher PHDI scores and hypertensive cases in this cohort may be a result of attributes of a plant-based diet with lower sodium content, increased potassium content, reduced oxidative stress, and beneficial effects on the microbiome [34].

Infants born to mothers with higher PHDI scores were less likely to be born SGA, possibly due to the well-established link between maternal nutritional status and placental function [35]. Recent research in a pregnant cohort revealed similar findings, with higher PHD scores associated with a lower likelihood of having a low birth weight [18,19]. Poor maternal nutrition can impair placental development and function, hindering the transfer of nutrients and oxygen to the fetus and increasing risk of intrauterine growth restriction and SGA [36]. In addition, hypertensive disorders in pregnancy such as PIH and preeclampsia can impair placental function and are associated with higher rates of SGA births [37]. Notably, adherence to the PHD appears to have a protective effect against these conditions, which may explain the reduced likelihood of SGA among those with higher PHDI scores [37]. These findings suggest that women in the “Low PHDI” group may have been less likely to meet the nutritional demands necessary to support optimal placental function, thereby increasing the risk of delivering a SGA infant.

The “High PHDI” group had significantly higher breastfeeding initiation rates postpartum. Higher breastfeeding initiation rates are often associated with higher education; however, no significant difference in third-level education was observed between the “High PHDI” and “Low PHDI” groups [38,39]. This suggests that the higher breastfeeding rates in the “High PHDI” group may be influenced by other factors such as a greater awareness of the environmental benefits of breastfeeding over formula feeding. The production of artificial milk formula contributes to increased greenhouse gas emissions, water pollution, and resource waste, making it misaligned with the environmental principles of the PHD [40]. Given that women in the “High PHDI” group followed more sustainable dietary behaviors aligned with the PHD, it is plausible that their commitment to environmentally conscious food choices extends beyond their own food choices, influencing infant feeding decisions as well.

The link between maternal dietary patterns and breastfeeding behavior remains largely underexplored. One study found that a more proinflammatory diet was associated with lower odds of breastfeeding [41]. In contrast, the PHD promotes a diet rich in anti-inflammatory components and is associated with more positive breastfeeding behaviors [41,42]. One possible explanation is the influence of maternal metabolic health on lactation. Adherence to healthier diets during pregnancy, such as the Mediterranean Diet, can positively impact metabolic function [43]. Maintaining a good metabolic profile in pregnancy, such as good insulin sensitivity, may support the mammary glands’ ability to produce milk effectively [44]. Therefore, adherence to a healthier dietary pattern during pregnancy, such as the PHD, could improve metabolic function, potentially enhancing early milk production and thereby increasing the likelihood of breastfeeding.

Our findings strengthen the evidence for healthcare professionals to advise women to follow the PHD during pregnancy and this advice could be tailored specifically to high-risk women with a raised BMI. Public health policies should continue to promote sustainable diets in pregnancy alongside regular supplementation.

The EAT-Lancet reference diet has been explored in relation to chronic diseases such as cancer, cardiovascular disease, and diabetes with most studies targeting the general population [[45], [46], [47]]. Pregnant women with OVOB, particularly in resource-limited settings, are at increased risk of both undernutrition and diet-related conditions [48]. Further research is needed to optimize sustainable and health-promoting diets for at-risk populations. In addition, current PHD scoring methods vary considerably and were not developed with specific nutritional needs of pregnant women in mind. Future RCTs are necessary to strengthen and validate the evidence found in this study. Other studies could also explore the PHD in other high-risk populations, such as women at risk of preterm birth or diagnosed with GDM, as the PHD might play a protective role in these groups. It would also be interesting to examine the feasibility of the PHD in pregnancy and how this dietary pattern impacts women with a lower socioeconomic status.

This study is among the first to examine the association between the PHD and pregnancy outcomes in women with OVOB, a group already at heightened risk of adverse pregnancy outcomes. A key strength of this study includes the use of 3-d food diaries, which offer a more precise dietary assessment than food frequency questionnaires by improving portion size accuracy and reducing recall bias [4]. Additional strengths include detailed pregnancy outcome data and adjustment for relevant confounders.

However, the demographic homogeneity of the cohort may restrict the applicability of the findings to more diverse populations. Inconsistencies in how adherence to the PHD is measured and scored across different studies also complicate comparison. This study did not assess full adherence to the PHDI but instead classified participants based on median scores. The “High PHDI” group reflected moderate, not strict, adherence to the PHD guidelines. Participants were not instructed to follow the PHD, and dietary behaviors were observed rather than influenced. The single dietary assessments at 28 wk only capture a snapshot of pregnancy nutrition and not overall dietary patterns throughout pregnancy. Future intervention studies could look at intentional adherence to the PHD as an intervention to more effectively examine its role during pregnancy. The PHD was only investigated in late pregnancy and not throughout pregnancy, which limits understanding of how adherence and dietary patterns may change across trimesters.

Other limitations include the focus on a high-risk cohort, which is not comparable with general healthy populations; however, given that 50% of pregnancies in Ireland have a BMI >25, these data are applicable to a large proportion of the population [49,50]. The “High PHDI” and “Low PHDI” groups also differed significantly by intervention allocation (42.8% compared with 57.2%), with the intervention itself affecting dietary intake, which creates an imbalance that adjustment may not fully address. The cohort was also subject to potential selection bias as women recruited to randomized trials are typically more educated and thus may live more sustainable lifestyles. A large proportion of women did not provide dietary data in late pregnancy, which restricted our sample size and may result in bias; however, additional analyses revealed no differences in pregnancy outcomes between women with and without dietary data. Associations may be due to residual confounding, and results should be interpreted with caution. Associations with SGA and breastfeeding initiation in some cases met a 20% but not a 10% FDR threshold; given the exploratory nature of this analysis, these findings should be interpreted with particular caution pending confirmation in future studies.

In conclusion, this study highlights the potential benefits of the PHD in pregnancy, including reduced risk of preeclampsia, PIH, and SGA births, as well as increased breastfeeding initiation rates postpartum. These findings were observed in women with OVOB, a population at elevated risk of adverse pregnancy outcomes, highlighting the potential of the PHD to support maternal and neonatal health within at-risk groups. Given the rising popularity of sustainable diets, it is crucial that these diets are adapted for times of increased nutritional demands such as pregnancy, keeping key pregnancy nutrients in mind.

## Author Contribution

AD analysed the data and drafted the initial manuscript. SC advised on statistical analysis and contributed to the manuscript preparation. G.A.C and E.O.B were involved in the study design and contributed to the manuscript preparation. A.P.D was responsible dor the data management and curation. F.M.McA designed the study and had primary responsibility for the final content. All authors approved the final version of the manuscript.

## Data availability

The data used and/or analyzed in these studies are available from the corresponding author on reasonable request.

## Declaration of generative AI and AI-assisted technologies in the writing process

The author(s) declare that no generative AI or AI-assisted technologies were used in the writing of this manuscript.

## Funding

The research was supported by University College Dublin, the National Maternity Hospital medical fund, and Research Ireland under Grant No. 22-FFA-10302.

## Conflict of interest

The authors report no conflicts of interest.
