# Supplementary material for: Associations between the Planetary Health Diet in Late Pregnancy and Pregnancy Outcomes in Women with a Raised BMI: Insights from the PEARS Study
Source: Curr Dev Nutr. 2026 Sep 19;10(10):109540. doi: 10.1016/j.cdnut.2026.109540 (PMC13678075; doi:10.1016/j.cdnut.2026.109540)
Supplement: Supplementary file 1 [file mmc1.docx]

**Associations between the Planetary Health Diet in Late Pregnancy and Pregnancy Outcomes in Women with a Raised BMI: Insights from the PEARS study**

**Supplementary Material**

**Supplementary methods:**

**Eligibility criteria and the intervention**

Participants were randomised into either the diet and physical activity intervention group or control group with standard antenatal care. The intervention arm received a one-time education session at randomisation, focusing on the encouragement of low glycaemic index foods and appropriate carbohydrate portion sizes. This session was led by a research dietitian or nutritionist and was supplemented through a mobile phone application, email communications, and follow-up sessions conducted at 28-34 weeks’ gestation (1).

***Dietary Assessment in Late Pregnancy***

Participants recorded their food and beverage intake using household measures (e.g., teaspoons, tablespoons) or the weight provided on product packaging. Upon collection, food diaries were reviewed for completeness, and any unclear or insufficient entries were clarified directly with participants either in person or over the phone. Participants were also encouraged to include food packaging labels with nutritional information or provide photos of meals (taken before and after consumption) using a provided scale to help assess portion size visually. Data were entered to Nutritics Professional Nutrition Analysis Software, version 4.267, Research Edition (Nutritics, Dublin, Ireland, [www.nutritics.com](http://www.nutritics.com)), a cloud-based tool containing over 125,000 food items sourced from various international databases. Foods with nutrient composition data from McCance and Widdowson’s Composition of Foods Integrated Dataset (CoFID) 2015 were chosen. If data were not available in CoFID 2015, other sources were used in the following order: CoFID 2008, the Irish Food Composition Database (2009), and Nutritics-sourced foods, supplements, and additives. Data was entered by nutritionists and exported from Nutritics. Mean daily nutrient intakes were calculated at 28 weeks’ gestation.

**Assignment of PHDI scores:**

Adequacy components include foods such as whole grains, whole fruits (excludes fruit juice), non-starchy vegetables, nuts and seeds, legumes, and unsaturated oils. Moderation components include starchy vegetables, dairy, red and processed meat, poultry, eggs, fish, saturated oils and trans fats, and added sugar and fruit juice. Scores for the oils and sugars were based on percentage energy; all others were based on weight of each component consumed. As recommended by the EAT-Lancet Commission, legumes were divided into two subgroups (nonsoy legumes and soybean/soy foods), each weighted at 0.5 for the purpose of score derivation meaning a total possible score of 5 for each. All other components were assigned a score ranging from 0 to 10, contributing to a total possible score of 140. Intakes between the minimum and maximum thresholds were scored proportionately using continuous scoring.

| Supplementary Table 1. Maternal and Neonatal outcome definitions | |
| --- | --- |
| Outcome | **Definition/Diagnostic Criteria** |
| Preeclampsia | Defined based on the National Institute for Health and Care Excellence (NICE) guideline, as new onset hypertension (>140 mm Hg systolic or >90 mm Hg diastolic) presenting after 20 weeks and prior to 34 weeks with significant proteinuria. |
| Pregnancy-induced hypertension | The onset of hypertension after 20 weeks’ gestation without proteinuria. |
| Gestational Weight Gain & Gestational Weight Gain Criteria | The last measured weight taken before delivery was recorded from medical charts and used to compute total gestational weight gain. Each participant was assigned to a gestational weight gain criteria group (adequate/inadequate/excessive) based on the Institute of Medicine Guidelines (2). |
| Anaemia | Anaemia in late pregnancy was defined as haemoglobin levels <11 g/dL. |
| Placental weight and Breastfeeding initiation. | Placental weight and breastfeeding initiation were obtained from medical records. |
| Preterm delivery | Delivery <37 completed weeks. |
| Head circumference (cm), birthweight (g), low birthweight (≤ 2.5 kg), macrosomia (cut-offs; > 4kg, ≥4.5kg) | At delivery, neonates head circumference. length, and birthweight was recorded. |
| Birthweight centile | The Gestation Network’s Bulk Caluclator 6.2.3 UK was used to calculate birth weight centiles (1). |
| Small for gestational age | (birthweight <10^th^ centile) (3, 4) |
| Large for gestational age | (birthweight>90^th^ centile) |

| **Supplementary Table 2.** Assessment of non-linear associations between PHDI score and statistically significant outcomes. | | | | |
| --- | --- | --- | --- | --- |
| **Outcome** | **Linear R^2^** | **Logarithmic R^2^** | **Quadratic R^2^** | **Cubic R^2^** |
| **Preeclampsia/PIH** | 0.018 | 0.018 | 0.018 | 0.019 |
| **SGA** | 0.02 | 0.02 | 0.02 | 0.02 |
| **Breastfeeding Initiation** | 0.03 | 0.03 | 0.03 | 0.03 |

| Supplementary Table 3. Nutrient intakes for total cohort in late pregnancy. | | |
| --- | --- | --- |
|  | **N** | **Mean (SD) / Median (IQR)** |
| *Diet quality* |  |  |
| PHDI score | 319 | 93.13 (10.57) |
| *Macronutrients* |  |  |
| Energy (kcal/day) | 319 | 1752.29 (1516.69, 2019.67) |
| Carbohydrates (g/day) | 319 | 212.01 (178.9, 250.11) |
| Total sugars (g/day) | 319 | 76.33 (61.76, 97.85) |
| Protein (g/day) | 319 | 81.23 (19.13) |
| Fat (g/day) | 319 | 70.09 (56.68, 84.44) |
| Saturated Fat (g/day)  Unsaturated Fat (g/day)  Omega 6 (g/day)  Omega 3 (g/day) | 319  319  319  319 | 26.79 (20.15, 32.32)  25.02 (19.43, 30.68)  5.03 (3.76, 6.67)  0.88 (0.59, 1.43) |
| Fibre (g/day) | 319 | 20.49 (17.19, 24.52) |
| *Micronutrients* |  |  |
| Vitamin A (µg/day) | 319 | 773.56 (507.65, 1134.91) |
| Vitamin D (µg/day) | 319 | 2.82 (1.64, 4.44) |
| Vitamin E (mg/day) | 319 | 9.39 (7.24, 12.44) |
| Vitamin C (mg/day) | 319 | 82.9 (52.53, 124.93) |
| Vitamin B1 (Thiamin) (mg/day) | 319 | 1.61 (1.36, 1.96) |
| Vitamin B2 (Riboflavin) (mg/day) | 319 | 1.75 (1.37, 2.17) |
| Vitamin B3 (Niacin) (mg/day) | 319 | 38.11 (10.07) |
| Vitamin B6 (mg/day) | 319 | 2.02 (0.65) |
| Vitamin B9 (Folate) (µg DFE/day) | 319 | 276.19 (218.26, 377.07) |
| Vitamin B12 (µg/day) | 319 | 4.54 (3.31, 6) |
| Sodium (mg/day) | 319 | 2098.04 (1679.97, 2580.16) |
| Potassium (mg/day) | 319 | 2889.04 (665.22) |
| Calcium (mg/day) | 319 | 886.26 (718.71, 1109.87) |
| Magnesium (mg/day) | 319 | 286.34 (239.06, 340.11) |
| Iron (mg/day) | 319 | 11.09 (9.22, 13.35) |
| Zinc (mg/day) | 319 | 9.09 (7.57, 10.84) |
| Iodine (µg/day) | 319 | 138.02 (104.04, 182.01) |
| Selenium (µg/day) | 319 | 44.9 (35.31, 55.02) |
| Kcal= Kilocalories. G= Grams. mg= milligram. µg = microgram. RE/day= Retinol equivalents per day. DFE/day = dietary folate equivalents per day. | | |

| Supplementary Table 4. Associations between the Planetary Health Diet Index and energy and macronutrient intakes in late pregnancy. | | | | | | | | | | |
| --- | --- | --- | --- | --- | --- | --- | --- | --- | --- | --- |
|  | **Low PHDI (≤92.9)** | | **High PHDI (>92.9)** | | **P value** | **Model** | **High PHDI (compared to Low PHDI)** | | **Per 1-point increment in PHDI score** | |
|  | **N** | **Mean (SD) / Median (IQR)** | **N** | **Mean (SD) / Median (IQR)** |  |  | **ß (95%)** | **P value** | **ß (95%)** | **P value** |
| Energy (kcal/day) | 160 | 1760.00 (1502.13, 2086.38) | 159 | 1748.55 (1529.53, 2003.90) | 0.90 | Model 1  Model 2 | 23.20 (-71.07, 117.46)  -20.71 (-103.85, 62.43) | 0.93  0.62 | -0.33 (-4.79, 4.14)  -2.62 (-6.55, 1.32) | 0.89  0.19 |
| Carbohydrates (g/day) | 160 | 213.33 (175.80, 254.14) | 159 | 209.27 (180.93, 245.97) | 0.99 | Model 1  Model 2 | 1.69 (-10.43, 13.82)  -2.95(-13.94, 8.05) | 0.78  0.60 | -0.05 (-0.62, 0.52)  -0.29 (-0.81, 0.24) | 0.53  0.28 |
|  |  |  |  |  |  |  |  |  |  |  |
| Total sugars (g/day) | 160 | 74.35 (56.31, 98.0) | 159 | 77.43 (65.23, 97.85) | 0.30 | Model 1  Model 2 | 4.53 (-2.03, 11.10)  1.49 (-4.84, 7.82) | 0.18  0.64 | 0.21 (-0.10, 0524)  0.07 (-0.23, 0.37) | 0.18  0.66 |
|  |  |  |  |  |  |  |  |  |  |  |
| Protein (g/day) | 160 | 82.8 (19.39) | 159 | 79.64 (18.78) | 0.14 | Model 1  Model 2 | -2.73 (-7.01, 1.55)  -4.15 (-8.20, 0.10) | 0.21  0.05 | -0.11 (-0.31, 0.09)  -0.18 (-0.37, 0.01) | 0.29  0.06 |
|  |  |  |  |  |  |  |  |  |  |  |
| Fat (g/day) | 160 | 69.62 (55.31, 84.97) | 159 | 70.09 (57.39, 84.44) | 0.78 | Model 1  Model 2 | 3.05 (-2.12, 8.21)  0.8 (-3.97, 5.57) | 0.25  0.74 | 0.04 (-0.21, 0.28)  -0.09 (-0.31, 0.14) | 0.78  0.46 |
|  |  |  |  |  |  |  |  |  |  |  |
| Saturated fat (g/day) | 160 | 26.85 (18.92, 34.64) | 159 | 26.63 (20.35, 31.00) | 0.88 | Model 1  Model 2 | 0.46 (-1.76, 2.67)  -0.44 (-2.51, 1.63) | 0.69  0.68 | -0.02 (-0.13, 0.08)  -0.07 (-0.17, 0.03) | 0.69  0.17 |
|  |  |  |  |  |  |  |  |  |  |  |
| Unsaturated fat (g/day) | 160 | 24.77 (18.74, 29.96) | 159 | 25.15 (19.98, 31.30) | 0.33 | Model 1  Model 2 | 1.65 (-0.36, 3.66)  0.88 (-1.04, 2.79) | 0.11  0.37 | -0.01 (-0.08, 0.11)  -0.03 (-0.12, 0.06) | 0.77  0.52 |
| Omega 3 (g/day) | 160 | 0.98 (0.60, 1.55) | 159 | 0.84 (0.55, 1.36) | 0.2 | Model 1  Model 2 | -0.16 (-0.38, 0.06)  -0.20 (-0.43, 0.02) | 0.16  0.07 | -0.01 (-0.02, -0.002)  -0.02 (-0.03, -0.01) | 0.02  0.01 |
| Omega 6 (g/day) | 160 | 4.92 (3.62, 6.28) | 159 | 5.55 (3.97, 7.57) | 0.03 | Model 1  Model 2 | 1.04 (0.36, 1.73)  0.79 (0.10, 1.47) | 0.003  0.02 | 0.07 (0.04, 0.10)  0.06 (0.03, 0.09) | <0.001  <0.001 |
| Fibre (g/day) | 160 | 18.46 (14.85, 21.65) | 159 | 22.85 (18.99, 27.15) | <0.001 | Model 1  Model 2 | 4.98 (3.72, 6.25)  4.51 (3.29, 5.72) | <0.001  <0.001 | 0.29 (0.23, 0.34)  0.26 (0.21, 0.32) | <0.001  <0.001 |
| ^a^Model 1 is adjusted for intervention. ^b^Model 2 is adjusted for study allocation group (intervention, yes/no), maternal BMI (continuous kg/m^2^) at baseline (14-16 weeks’ gestation), physical activity (continuous Metabolic Equivalents of Task (METs)/day), HP index (continuous score) and dietary misreporting status (under reporter, yes/no). ß (95%) = Beta Coefficient (95% Confidence Interval). | | | | | | | | | | |

| Supplementary Table 5. Associations between the Planetary Health Diet Index and micronutrient intakes in late pregnancy. | | | | | | | | | | |
| --- | --- | --- | --- | --- | --- | --- | --- | --- | --- | --- |
|  | Low PHDI | | High PHDI | | P value | Model | High PHDI | | Per 1-point increment in PHDI score | |
|  | N | Median (IQR) | N | Median (IQR) |  |  | ß (95%) | P value | ß (95%) | P value |
| Vitamin A (µg/day) | 160 | 723.2 (466.51, 1107.02) | 159 | 837.5 (547.5, 1253.28) | 0.039 | ^a^Model 1  ^b^Model 2 | 110.99 (-5.14, 227.11)  79.46 (-37.54, 196.47) | 0.06  0.18 | 8.01 (2.56, 13.47)  6.34 (0.82, 11.86) | 0.004  0.02 |
| Vitamin D (µg/day) | 160 | 3.06 (1.97, 4.68) | 159 | 2.41 (1.39, 3.95) | 0.006 | ^a^Model 1  ^b^Model 2 | -0.91 (-1.47, -0.34)  -0.94 (-1.52, -0.37) | 0.002  0.001 | -0.05 (-0.08, -0.02)  -0.05 (-0.08, -0.03) | <0.001  <0.001 |
| Vitamin E (mg/day) | 160 | 8.98 (7.02, 11.82) | 159 | 9.58 (7.51, 13.2) | 0.08 | ^a^Model 1  ^b^Model 2 | 0.81 (-0.06, 1.68)  0.48 (-0.38, 1.33) | 0.07  0.27 | 0.07 (0.03, 0.11)  0.05 (0.01, 0.09) | 0.001  0.01 |
| Vitamin C (mg/day) | 160 | 73.86 (48.39, 113.65) | 159 | 97.33 (65.34, 134) | <0.001 | ^a^Model 1  ^b^Model 2 | 17.45 (5.45, 29.45)  14.24 (2.05, 26.42) | 0.004  0.02 | 1.16 (0.60, 1.72)  1.01 (0.44, 1.58) | <0.001  <0.001 |
| Vitamin B1 (Thiamin) (mg/day) | 160 | 1.56 (1.31, 1.95) | 159 | 1.68 (1.38, 1.96) | 0.08 | ^a^Model 1  ^b^Model 2 | 0.16 (-0.03, 0.35)  0.13 (-0.06, 0.32) | 0.09  0.17 | 0.01 (0.01, 0.02)  0.01 (0.003, 0.02) | 0.003  0.01 |
| Vitamin B2 (Riboflavin) (mg/day) | 160 | 1.72 (1.38, 2.20) | 159 | 1.75 (1.37, 2.09) | 0.84 | ^a^Model 1  ^b^Model 2 | -0.01 (-0.14, 0.12)  -0.05 (-0.17, 0.08) | 0.89  0.48 | 0.00 (-0.01, 0.01)  -0.002 (-0.01, 0.004) | 0.90  0.50 |
| Vitamin B3 (Niacin) (mg/day) | 160 | 38.92 (10.42) | 159 | 37.29 (9.67) | 0.012 | ^a^Model 1  ^b^Model 2 | -1.90 (-3.43, -0.36)  -2.24 (-3.76, -0.71) | 0.02  0.004 | -0.12 (-0.19, -0.04)  -0.13 (-0.20, -0.06) | 0.002  <0.001 |
| Vitamin B6 (Pyridoxine) (mg/day) | 160 | 2.05 (0.73) | 159 | 1.98 (0.57) | 0.25 | ^a^Model 1  ^b^Model 2 | -0.08 (-0.23, 0.07)  -0.13 (-0.27, 0.02) | 0.28  0.08 | -0.001 (-0.01, 0.01)  -0.004 (-0.01, 0.003) | 0.71  0.29 |
| Vitamin B9 (Folate) (µg DFE/day) | 160 | 255.50 (200.11, 378.23) | 159 | 285.12 (232.26, 377.07) | 0.031 | ^a^Model 1  ^b^Model 2 | 16.46 (-18.71, 51.62)  13.42 (-22.26, 49.09) | 0.36  0.46 | 1.87 (0.22, 3.53)  1.77 (0.08, 3.45) | 0.03  0.04 |
| Vitamin B12  (Cobalamin) (µg/day) | 160 | 4.97 (3.49, 6.04) | 159 | 4.22 (3.18, 5.8) | 0.043 | ^a^Model 1  ^b^Model 2 | -0.54 (-1.04, -0.03)  -0.66 (-1.17, -0.16) | 0.04  0.01 | -0.04 (-0.07, -0.02)  -0.05 (-0.07, -0.03) | <0.001  <0.001 |
| Sodium (mg/day) | 160 | 2117.83 (1704.33, 2666.17) | 159 | 2085.41 (1633.40, 2464.62) | 0.49 | ^a^Model 1  ^b^Model 2 | -7.64 (-163.74, 148.47)  -39.95 (-192.10, 112.19) | 0.92  0.61 | -0.44 (-7.83, 6.95)  -2.28 (-9.49, 4.94) | 0.91  0.54 |
| Potassium (mg/day) | 160 | 2816.73 (661.32) | 159 | 2961.80 (663.25) | 0.05 | ^a^Model 1  ^b^Model 2 | 151.36 (2.49, 300.24)  83.55 (-54.96, 222.07) | 0.05  0.24 | 8.89 (1.87, 15.91)  5.59 (-0.97, 12.14) | 0.01  0.10 |
| Calcium (mg/day) | 160 | 866.21 (710.99, 1112.26) | 159 | 937.10 (728.65, 1109.87) | 0.22 | ^a^Model 1  ^b^Model 2 | 50.66 (-21.03, 122.34)  26.95 (-42.04, 95.93) | 0.17  0.44 | 2.99 (-0.40, 6.38)  1.84 (-1.43, 5.11) | 0.08  0.27 |
| Magnesium (mg/day) | 160 | 267.61 (219.85, 313.39) | 159 | 302.67 (252.82, 368.28) | <0.001 | ^a^Model 1  ^b^Model 2 | 47.38 (30.29, 64.47)  38.61 (22.56, 54.67) | <0.001  <0.001 | 2.82 (2.03, 3.60)  2.40 (1.66, 3.14) | <0.001  <0.001 |
| Iron (mg/day) | 160 | 10.84 (8.53, 12.71) | 159 | 11.62 (9.58, 14.12) | 0.003 | ^a^Model 1  ^b^Model 2 | 1.18 (0.48, 1.88)  0.95 (0.28, 1.62) | 0.001  0.006 | 0.06 (0.03, 0.09)  0.05 (0.02, 0.08) | <0.001  0.004 |
| Zinc (mg/day) | 160 | 8.85 (2.54) | 159 | 9.89 (2.46) | <0.001 | ^a^Model 1  ^b^Model 2 | 1.11 (0.55, 1.67)  0.90 (0.37, 1.43) | <0.001  <0.001 | 0.06 (0.03, 0.08)  0.05 (0.02, 0.07) | <0.001  <0.001 |
| Iodine (µg/day) | 160 | 138.97 (101.30, 191.66) | 159 | 133.62 (104.92, 173.91) | 0.32 | ^a^Model 1  ^b^Model 2 | -7.98 (-22.28, 6.33)  -11.31 (-25.59, 2.97) | 0.27  0.12 | -0.06 (-0.74, 0.62)  -0.23 (-0.90, 0.46) | 0.85  0.52 |
| Selenium (µg/day) | 160 | 47.93 (35.71, 58.36) | 159 | 41.93 (34.57, 51.82) | 0.011 | ^a^Model 1  ^b^Model 2 | -5.44 (-9.30, -1.58)  -6.15 (-10.06, -2.25) | 0.006  0.002 | -0.29 (-0.47, -0.11)  -0.33 (-0.52, -0.15) | 0.002  <0.001 |
| ^a^Model 1 is adjusted for the study allocation group (intervention, yes/no). ^b^Model 2 is adjusted for study allocation group (intervention, yes/no), maternal BMI (continuous kg/m^2^) at baseline (14-16 weeks’ gestation), physical activity (continuous Metabolic Equivalents of Task (METs)/day), HP index (continuous score), and dietary misreporting status (under reporter, yes/no).  ß (95%)= Beta Coefficient (95% Confidence Interval) | | | | | | | | | | |

| **Supplementary Table 6. Adjusted logistic regression for breastfeeding, PIH and preeclampsia, and SGA vs each one-point increment in PHDI component.** | | | | |
| --- | --- | --- | --- | --- |
| Pregnancy outcome | PHDI Component | Model | Per 1 point increment increase in PHDI component | |
|  |  |  | Odds Ratio | P value |
| Preeclampsia and /PIH | Wholegrains score | ^a^Model 1  ^b^Model 2 | 0.93 (0.82, 1.05)  0.92 (0.81, 1.04) | 0.24  0.19 |
|  | Whole fruit score | ^a^Model 1  ^b^Model 2 | 0.90 (0.78, 1.03)  0.89 (0.77, 1.03) | 0.14  0.12 |
|  | Non-Starchy vegetables score | ^a^Model 1  ^b^Model 2 | 0.88 (0.72, 1.09)  0.87 (0.70, 1.08) | 0.25  0.20 |
|  | Nuts seeds score | ^a^Model 1  ^b^Model 2 | 0.71 (0.44, 1.15)  0.68 (0.41, 1.14) | 0.16  0.14 |
|  | Non soy legumes score | ^a^Model 1  ^b^Model 2 | 0.81 (0.62, 1.06)  0.80 (0.61, 1.05) | 0.81  0.11 |
|  | Soy score | ^a^Model 1  ^b^Model 2 | 0.02 (0,0)  0.02 (0,0) | 1.0  1.0 |
|  | Overall Legume Score | ^a^Model 1  ^b^Model 2 | 0.64 (0.38, 1.09)  0.63 (0.36, 1.08) | 0.10  0.09 |
|  | Unsaturated oils score | ^a^Model 1  ^b^Model 2 | 4301936704 (0,0)  2632213508 (0,0) | 1.0  1.0 |
|  | Starchy vegetables score | ^a^Model 1  ^b^Model 2 | 0.91 (0.78, 1.06)  0.89 (0.76, 1.04) | 0.21  0.15 |
|  | Dairy Score | ^a^Model 1  ^b^Model 2 | 0.81 (0.45, 1.47)  0.80 (0.44, 1.44) | 0.49  0.45 |
|  | Red meat score | ^a^Model 1  ^b^Model 2 | 0.94 (0.78, 1.14)  0.93 (0.77, 1.13) | 0.52  0.47 |
|  | Poultry Scores | ^a^Model 1  ^b^Model 2 | 0.99 (0.88, 1.10)  0.99 (0.88, 1.10) | 0.82  0.79 |
|  | Egg Score | ^a^Model 1  ^b^Model 2 | 1.13 (0.87, 1.48)  1.13 (0.86, 1.49) | 0.35  0.37 |
|  | Fish Score | ^a^Model 1  ^b^Model 2 | 1.02 (0.90, 1.15)  1.03 (0.90, 1.17) | 0.82  0.68 |
|  | Saturated fat Score | ^a^Model 1  ^b^Model 2 | 0.85 (0.54, 1.36)  0.85 (0.54, 1.35) | 0.50  0.50 |
|  | Added sugar score | ^a^Model 1  ^b^Model 2 | 0.79 (0.62, 1.01)  0.78 (0.61, 1.01) | 0.06  0.06 |
| SGA | Wholegrains score | ^a^Model 1  ^b^Model 2 | 0.90 (0.79, 1.01)  0.88 (0.77, 1.0) | 0.07  0.04 |
|  | Whole fruit score | ^a^Model 1  ^b^Model 2 | 0.91 (0.79, 1.04)  0.90 (0.78, 1.04) | 0.15  0.13 |
|  | Non-Starchy vegetables score | ^a^Model 1  ^b^Model 2 | 0.86 (0.69, 1.07)  0.85 (0.68, 1.07) | 0.17  0.16 |
|  | Nuts seeds score | ^a^Model 1  ^b^Model 2 | 0.70 (0.42, 1.17)  0.67 (0.39, 1.14) | 0.17  0.14 |
|  | Non soy legumes score | ^a^Model 1  ^b^Model 2 | 0.79 (0.60, 1.04)  0.79 (0.59, 1.05) | 0.10  0.11 |
|  | Soy score | ^a^Model 1  ^b^Model 2 | 0.02 (0,0)  0.02 (0,0) | 1.0  1.0 |
|  | Overall Legume Score | ^a^Model 1  ^b^Model 2 | 0.61 (0.35, 1.07)  0.62 (0.34, 1.08) | 0.08  0.09 |
|  | Unsaturated oils score | ^a^Model 1  ^b^Model 2 | ----  ---- | 1.0  1.0 |
|  | Starchy vegetables score | ^a^Model 1  ^b^Model 2 | 1.13 (0.92, 1.38)  1.12 (0.91, 1.38) | 0.25  0.31 |
|  | Dairy Score | ^a^Model 1  ^b^Model 2 | 0.64 (0.41, 1.0)  0.64 (0.41, 1.01) | 0.05  0.05 |
|  | Red meat score | ^a^Model 1  ^b^Model 2 | 0.94 (0.77, 1.14)  0.92 (0.75, 1.13) | 0.52  0.42 |
|  | Poultry Scores | ^a^Model 1  ^b^Model 2 | 0.99 (0.89, 1.11)  0.99 (0.88, 1.11) | 0.91  0.81 |
|  | Egg Score | ^a^Model 1  ^b^Model 2 | 0.94 (0.78, 1.13)  0.94 (0.77, 1.14) | 0.51  0.53 |
|  | Fish Score | ^a^Model 1  ^b^Model 2 | 0.97 (0.87, 1.09)  0.97 (0.86, 1.09) | 0.59  0.56 |
|  | Saturated fat Score | ^a^Model 1  ^b^Model 2 | 0.71 (0.46, 1.11)  0.72 (0.46, 1.13) | 0.13  0.16 |
|  | Added sugar score | ^a^Model 1  ^b^Model 2 | 0.91 (0.69, 1.19)  0.92 (0.70, 1.20) | 0.47  0.52 |
| Breastfeeding initiation | Wholegrains score | ^a^Model 1  ^c^Model 2 | 1.10 (1.10, 1.17)  1.06 (0.98, 1.14) | 0.01  0.14 |
|  | Whole fruit score | ^a^Model 1  ^c^Model 2 | 1.12 (1.04, 1.21)  1.10 (1.01, 1.19) | 0.002  0.02 |
|  | Non-Starchy vegetables score | ^a^Model 1  ^c^Model 2 | 1.29 (1.15, 1.46)  1.25 (1.10, 1.42) | <0.001  <0.001 |
|  | Nuts seeds score | ^a^Model 1  ^c^Model 2 | 1.24 (1.04, 1.47)  1.16 (0.97, 1.39) | 0.02  0.10 |
|  | Non soy legumes score | ^a^Model 1  ^c^Model 2 | 1.01 (0.92, 1.10)  0.98 (0.89, 1.08) | 0.91  0.70 |
|  | Soy score | ^a^Model 1  ^c^Model 2 | 102.26 (0,0)  83.88 (0,0) | 1.0  1.0 |
|  | Overall Legume Score | ^a^Model 1  ^c^Model 2 | 1.05 (0.88, 1.25)  0.99 (0.82, 1.20) | 0.62  0.90 |
|  | Unsaturated oils score | ^a^Model 1  ^c^Model 2 | 0 (0, 0)  0 (0, 0) | 1.0  1.0 |
|  | Starchy vegetables score | ^a^Model 1  ^c^Model 2 | 1.15 (1.06, 1.25)  1.13 (1.03, 1.24) | <0.001  0.01 |
|  | Dairy Score | ^a^Model 1  ^c^Model 2 | 1.02 (0.71, 1.45)  1.05 (0.70, 1.56) | 0.93  0.83 |
|  | Red meat score | ^a^Model 1  ^c^Model 2 | 1.03 (0.93, 1.15)  1.02 (0.90, 1.14) | 0.53  0.81 |
|  | Poultry Scores | ^a^Model 1  ^c^Model 2 | 0.98 (0.93, 1.04)  0.97 (0.91, 1.04) | 0.55  0.43 |
|  | Egg Score | ^a^Model 1  ^c^Model 2 | 0.95 (0.84, 1.06)  0.95 (0.84, 1.07) | 0.33  0.41 |
|  | Fish Score | ^a^Model 1  ^c^Model 2 | 0.92 (0.86, 0.98)  0.93 (0.86, 1.0) | 0.02  0.04 |
|  | Saturated fat Score | ^a^Model 1  ^c^Model 2 | 0.83 (0.64, 1.08)  0.85 (0.63, 1.14) | 0.17  0.28 |
|  | Added sugar score | ^a^Model 1  ^c^Model 2 | 1.07 (0.92, 1.24)  1.09 (0.92, 1.29) | 0.39  0.32 |
| Abbreviations: PHDI= Planetary Health Diet Index; SGA, Small for Gestational Age; PIH, Pregnancy-induced hypertension; ^a^Model 1 is adjusted for the study allocation group (intervention, yes/no). ^b^Model 2 is adjusted for study allocation group (intervention, yes/no), maternal BMI (continuous kg/m^2^) at baseline (14-16 weeks’ gestation), physical activity (continuous Metabolic Equivalents of Task (METs)/day), and HP index (continuous score). ^c^Model 2 is additionally adjusted for education status (third level education yes/no). N for each outcome reflects participants with complete data for that specific measure. Sample sizes vary for each outcome and outcome were collected from different sources including hospital records, self-report questionnaires, postnatal visits, and availability differed by outcome. | | | | |

| Supplementary Table 7. Differences in pregnancy outcomes between those who provided dietary and those with missing dietary data in the PEARS study. | | | | | |
| --- | --- | --- | --- | --- | --- |
|  | No Dietary Data | | Dietary Data | | P value |
|  | N | N (%)/ Median (IQR)/Mean (SD) | N | N (%)/ Median (IQR)/Mean (SD) |  |
| ^c^Preeclampsia (yes) | 207 | 4 (1.9%) | 282 | 8 (2.8%) | 0.57 |
| ^c^PIH | 208 | 10 (4.8%) | 283 | 10 (3.5%) | 0.50 |
| ^c^Preeclampsia and/or PIH | 208 | 14 (6.7%) | 282 | 18 (6.4%) | 1.0 |
| ^a^Final maternal weight before delivery (kg) | 152 | 90.55 (83.03, 98.30) | 207 | 89 (82.6, 99.3) | 0.78 |
| ^b^Gestational weight gain (kg)  ^c^Adequate  ^c^Inadequate  ^c^Excessive | 152 | 12.08 (5.66)  41 (27%)  24 (15.8%)  87 (57.2%) | 204 | 11.86 (5.60)  54 (26.5%)  33 (16.2%)  117 (57.4%) | 0.72  0.99 |
| ^c^Anaemia in late pregnancy (yes) | 100 | 26 (26%) | 107 | 24 (22.4%) | 0.63 |
| ^a^Placental Weight (g) | 200 | 667.50 (565.25, 770) | 271 | 650 (568, 743) | 0.21 |
| ^c^Gestation at delivery  <37 weeks  ≥37 weeks | 227 | 11 (4.8%)  216 (95.2%) | 314 | 11 (3.5%)  303 (96.5%) | 0.51 |
| ^a^Head Circumference (cm) | 213 | 35.4 (34.5, 36) | 286 | 35.0 (34, 36) | 0.05 |
| ^a^Birthweight (g) | 229 | 3700 (3370, 3950) | 317 | 3600 (3317.50, 3970) | 0.21 |
| ^c^Low Birthweight (≤2.5kg) | 229 | 9 (3.9%) | 317 | 6 (1.9%) | 0.19 |
| ^c^Macrosomic (>4kg) | 229 | 47 (20.5%) | 317 | 71 (22.4%) | 0.67 |
| ^c^Macrosomic (≥4.5kg) | 229 | 11 (4.8%) | 317 | 12 (3.8%) | 0.67 |
| ^a^Birthweight centile | 210 | 55.65 (29.18, 80.23) | 302 | 51.40 (27.83, 74.98) | 0.39 |
| ^c^Birthweight <10^th^ centile (SGA) | 210 | 17 (8.1%) | 302 | 18 (6.0%) | 0.38 |
| ^c^Birthweight > 90^th^ centile (LGA) | 210 | 27 (12.9%) | 302 | 31 (10.3%) | 0.40 |
| *^c^Breastfeeding Initiation after Delivery*  Any BM exposure  Formula | 226 | 152 (67.3%)  74 (32.7%) | 315 | 222 (70.5%)  93 (29.5%) | 0.45 |
| ^a^Non-normal distribution – compared using the Mann–Whitney U test. Expressed as median (interquartile range, IQR).  ^b^Normal distribution – compared using independent samples t-tests. Expressed as mean (standard deviation, SD).  ^c^Categorical variables – compared with χ ^2^ tests. Expressed as n (% denominator).  SGA, Small for Gestational Age; LGA, Large for Gestational Age; PIH, Pregnancy-induced hypertension; GWG, Gestational Weight Gain. | | | | | |

**Supplementary References**

1.	Kennelly MA, Ainscough K, Lindsay KL, O'Sullivan E, Gibney ER, McCarthy M, et al. Pregnancy Exercise and Nutrition With Smartphone Application Support: A Randomized Controlled Trial. Obstet Gynecol. 2018;131(5):818-26.

2.	Gilmore LA, Redman LM. Weight gain in pregnancy and application of the 2009 IOM guidelines: toward a uniform approach. Obesity (Silver Spring). 2015;23(3):507-11.

3.	Saenger P, Czernichow P, Hughes I, Reiter EO. Small for Gestational Age: Short Stature and Beyond. Endocrine Reviews. 2007;28(2):219-51.

4.	Clayton PE, Cianfarani S, Czernichow P, Johannsson G, Rapaport R, Rogol A. Management of the Child Born Small for Gestational Age through to Adulthood: A Consensus Statement of the International Societies of Pediatric Endocrinology and the Growth Hormone Research Society. The Journal of Clinical Endocrinology & Metabolism. 2007;92(3):804-10.
